# Safety and Efficacy of Transcatheter Occlusion of Perimembranous Ventricular Septal Defect with Aortic Valve Prolapse: A Six-Year Follow-Up Study

**DOI:** 10.1155/2021/6634667

**Published:** 2021-03-18

**Authors:** Wenqian Zhang, Chaojie Wang, Shenrong Liu, Lingmei Zhou, Junjie Li, Jijun Shi, Mingyang Qian, Shushui Wang, Yu-Mei Xie, Zhiwei Zhang

**Affiliations:** ^1^Graduate School, The Second School of Clinical Medicine, Southern Medical University, Guangzhou, China; ^2^Department of Cardiac Pediatrics, Guangdong Provincial Cardiovascular Institute, Guangdong Provincial People's Hospital, Guangdong Academy of Medical Sciences, Guangzhou, China; ^3^Department of Cardiovascular Surgery, Guangdong Cardiovascular Institute, Guangdong Provincial General Hospital, Guangdong Academy of Medical Sciences, Guangzhou, China

## Abstract

**Background:**

With the rapid development of transcatheter techniques and instruments, transcatheter occlusion for patients with perimembranous ventricular septal defect (pVSD) and aortic valve prolapse (AVP) was constantly being tried, while the efficacy and safety of pVSD with AVP remain controversial.

**Objective:**

The aim of this study was to evaluate long-term efficacy and safety of transcatheter occlusion of pVSD with AVP.

**Methods:**

We retrospectively analyzed 164 children with pVSD and AVP who underwent transcatheter occlusion between January 2013 and November 2014. AVP was divided into 3 degrees according to right coronary leaflet morphology at end-diastole during aortic root angiography. Patient demographic and clinical data were collected.

**Results:**

There were 97 males and 67 females (median age, 40.0 (30.0–62.7) months; average weight, 16.94 ± 9.02 kg). Mild (*n* = 63), moderate (*n* = 89), and severe (*n* = 12) AVP success rates were 93.7%, 89.9%, and 58.3%, respectively. Immediately after procedure, there was no new-onset aortic regurgitation (AR) above trivial degree, residual shunt above mild degree, or complications requiring medication or operation, except for 1 patient who developed transient complete atrioventricular block. During follow-up, 1 mild AVP patient aggravated from mild to moderate AR and 1 moderate AVP patient aggravated from trivial to moderate AR. The new-onset AR in mild, moderate, and severe AVP was 2%, 1.8%, and 20%, respectively. AR disappeared in 17 patients. Residual shunt occurred in 9 patients after procedure, 4 of which disappeared during the follow-up period. No serious complications occurred in any patient during follow-up. Five-year cardiovascular event-free survival rates for mild, moderate, and severe AVP were 89.6%, 94.5%, and 80.0%, respectively.

**Conclusion:**

Transcatheter occlusion of pVSD with mild and moderate AVP has a high success rate and few complications, which is safe and effective in long-term follow-up. Transcatheter occlusion of pVSD with severe AVP has low success rates and high AR incidence. Therefore, transcatheter occlusion of pVSD with AVP is recommended for mild to moderate, but not severe, AVP.

## 1. Introduction

Ventricular septal defect (VSD) is the most common congenital heart disease excluding bicuspid aortic valve, accounting for approximately 40% of cases [1]; perimembranous ventricular septal defect (pVSD) is the most common type of VSD. Owing to the lack of anatomical support to the aortic valve and the Venturi effect of ventricular septal defect shunt, the right coronary leaflet tends to prolapse, with a reported prevalence of 7.8%–24.8% [2–5]. Traditionally, surgical treatment is needed for patients with VSD and AVP, but it is often accompanied by bleeding, wound infection, diaphragm paralysis, and chylothorax [6]. However, with the rapid development of transcatheter techniques and instruments, transcatheter occlusion for patients with pVSD and aortic valve prolapse (AVP) was constantly being tried [7, 8], while the efficacy and safety of pVSD with AVP remain controversial. This study retrospectively evaluated the long-term efficacy and safety of transcatheter occlusion of pVSD complicated with AVP in children.

## 2. Methods

### 2.1. Patients

We retrospectively reviewed 164 patients with pVSD with AVP who underwent transcatheter occlusion of VSD between January 2013 and November 2014 at Guangdong Provincial People's Hospital. All patients fulfilled the following criteria: age ≥2 years and pVSD with AVP diagnosed by cardiac catheterization. Exclusion criteria were as follows: (i) other congenital heart diseases requiring transcatheter occlusion, such as atrial septal defect, patent ductus arteriosus, patent foramen ovale, etc.; (ii) other heart malformations requiring surgical treatment or residual shunt after VSD surgical repair; and (iii) bicuspid aortic valve. Patients' general demographic and clinical data were reviewed, including electrocardiogram, transthoracic echocardiography examinations, and catheterization procedure, etc.

This study was approved by the research ethics committee of Guangdong Provincial People's Hospital (No. GDREC2016427A). Written informed consent was obtained from the guardians of all patients before the procedure.

### 2.2. Transcatheter Procedure

**Figure 1:**
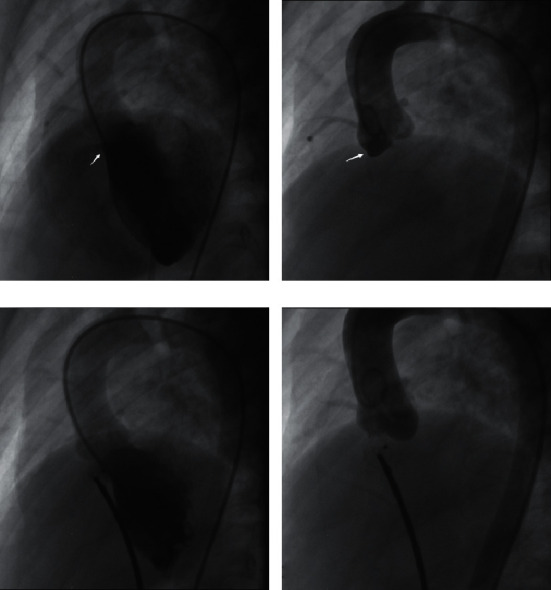
(a) LV angiography (60 left anterior oblique and 20 cranial) using a pigtail catheter to determine the location, shape, and size of VSD (white arrows). (b) Aortic root angiography (60 left anterior oblique and 20 cranial) demonstrating moderate AVP (white arrows). (c) Before the occluder released, LV angiography showed no residual shunt and satisfying position and shape of the occluder. (d) Aortic root angiography confirming the absence of AR prior to release of the occluder (LV = left ventricle; VSD = ventricular septal defect; AVP = aortic valve prolapse; AR = aortic regurgitation).

The transcatheter procedure was performed as previously described [9, 10]. Aortic root and left ventricle angiography were performed (60 left anterior oblique and 20 cranial) in all patients to determine pVSD location, shape, size, and relationship with the adjacent aortic valve, and AVP and aortic regurgitation (AR) grades. Appropriate occluders were selected according to the shape and size of the VSD, as determined by combined angiography with intra-procedural transthoracic echocardiography. Symmetrical VSD occluders (ShenZhen Lifetech Scientific Co., Ltd, China), eccentric VSD occluders (ShenZhen Lifetech Scientific Co., Ltd, China), and the Amplatzer Duct Occluder II (ADO-II; AGA Medical Corporation, Abbott Park, IL, USA) were used. When VSD is close to the aortic valve, an ADO-II occluder or eccentric occluder can be chosen. Symmetrical occluders are suitable for VSD with aneurysms. In our experience, occluders were selected that were 1–3 mm larger than the VSD size measured by left ventricular angiography. Before the device was released, the shape and position of the occluder, residual shunt, and valvular regurgitation were reassessed by aortic root angiography and left ventricle angiography (40–45 left anterior oblique and 20 cranial), and transthoracic echocardiography (Figure 1). A successful procedure was defined as the successful closure of pVSD with the device at an appropriate location, without more than mild residual shunt, and no surgery was required.

### 2.3. Grading Method

**Figure 2:**
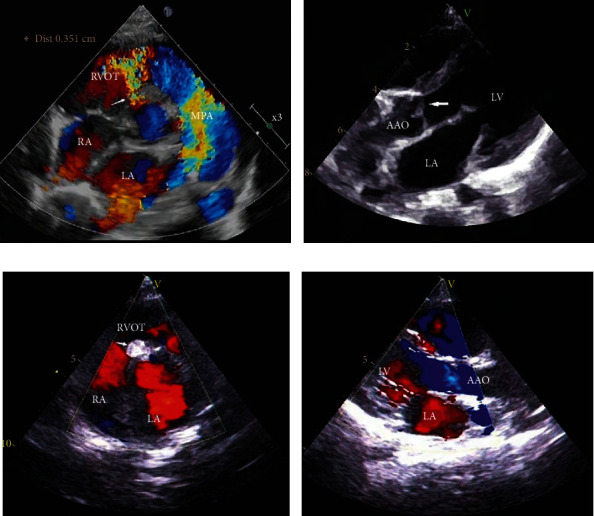
(a) Transthoracic echocardiography (TTE) showing the location of perimembranous ventricular septal defect and color Doppler image demonstrating a left to right jet through the defect (white arrows). (b) Moderate AVP is evident (white arrows). (c) TTE demonstrating optimal occluder position and absence of residual shunt after release of the occluder (white arrows). (d) After successfully implanting VSD occluder, parasternal long-axis TTE confirms no AR (LA = left atrium; RA = right atrium; RVOT = right ventricular outflow tract; MPA = main pulmonary artery; LV = left ventricle; AVP = aortic valve prolapse; AR = aortic regurgitation; VSD = ventricular septal defect; AAO = ascending aorta).

AVP was divided into 3 degrees according to the morphology of the right coronary leaflet at the end of diastole during angiography: mild (right coronary cusp buckling down the left ventricular outflow tract), moderate (right coronary cusp and its sinus prolapse into the VSD, partial blocking of the VSD with or without AR), and severe (cusp and sinus prolapse into the VSD within the right ventricular outflow tract, blocking most defects, which can lead to AR) [9, 11] (Figure 2).

### 2.4. Follow-Up

All patients received oral aspirin (3–5 kg/kg/d) for 6 months after the procedure. Patients who had no complications after transthoracic echocardiography, electrocardiogram, and chest x-ray examination were discharged 48 hours post-procedure.

Clinical evaluation, electrocardiogram, and transthoracic echocardiography were performed at 1, 3, 6, and 12 months after discharge, and thereafter yearly, to evaluate AVP, AR, position and shape of the occluder, residual shunt, tricuspid regurgitation, arrhythmia, etc. Cardiac adverse events were defined as more than moderate AR, new arrhythmias, residual shunts requiring surgical or transcatheter treatment, heart failure, cardiac arrest, and other complications requiring surgical treatment during follow-up.

AR was classified as trivial (AR jet reaching just beneath the aortic valve), mild (AR jet not exceeding the anterior mitral valve), moderate (AR jet reaching beyond the anterior cusp of the mitral valve but not reaching the left ventricular apex), and severe (AR jet reaching the left ventricular apex) by two-dimensional and color Doppler echocardiography in parasternal long-axis view [12–14].

### 2.5. Statistical Analysis

All statistical analyses were performed using SPSS Statistic version 25.0 (IBM Corp, Armonk, NY, USA). Categorical variables were expressed as numbers and percentages. Normally distributed data were expressed as mean ± standard deviation, and non-normally distributed data were expressed as median and interquartile range. Cumulative event-free survival was estimated using Kaplan–Meier analyses. *p* < 0.05 was considered statistically significant.

## 3. Results

### 3.1. Patients' Characteristics

A total of 164 patients with pVSD complicated with AVP were enrolled, including 97 male children (59.1%) and 67 female children (40.9%). The patients' demographic characteristics are described in Table 1. The median age and body weight at procedure were 40.00 (30.00–62.75) months and 16.94 ± 9.02 kg. Mean VSD size was 3.75 ± 1.35 mm, as evaluated by transthoracic electrocardiography. Mild AVP was found in 63 patients (38.4%), moderate AVP in 89 patients (54.3%), and severe AVP in 12 patients (23.6%). Catheterization data are shown in Table 2.

### 3.2. Successful Rate

Of the 164 patients who underwent transcatheter occlusion, the procedure was successful in 146. The success rates of mild, moderate, and severe AVP were 93.7%, 89.9%, and 58.3%, respectively, and the occluder sizes were 6.41 ± 1.40 mm, 6.52 ± 1.26 mm, and 6.00 ± 1.29 mm, respectively. The 18 patients in whom transcatheter occlusion failed ultimately underwent surgical repair of VSD.

In the 59 mild cases of AVP, 44 symmetrical VSD occluders, 13 eccentric VSD occluders, and 2 ADO-II occluders were used for closure. Of the 80 patients with moderate AVP, 50 symmetrical VSD occluders, 28 eccentric VSD occluders, and 2 ADO-II occluders were used. For the 7 severe AVP patients, 1 symmetrical VSD occluder, 4 eccentric VSD occluders, and 2 ADO-II occluders were used.

### 3.3. Progression of AR after Transcatheter Occlusion

In the mild AVP group, for the 8 patients with trivial or mild pre-procedural AR, 4 patients decreased or disappeared and 4 patients remained unchanged after procedure. At the end of follow-up, the degree of AR was increased to moderate in 1 (1.69%) patient, who had a mild AR after procedure. In 51 patients with mild AVP without AR before procedure, 11 developed trivial post-procedural AR. At the end of follow-up, 1 patient aggravated to mild AR, and 9 patients had trivial AR.

In the moderate AVP group, for the 25 patients with trivial or mild pre-procedural AR, 20 patients decreased or disappeared and 5 patients remained unchanged after procedure. At the end of follow-up, the degree of AR was increased to moderate in 1 (1.25%) patient, who had a trivial AR after procedure. In 55 patients with moderate AVP without AR before procedure, 13 developed trivial post-procedural AR. At the end of follow-up, 1 patient (1.8%) aggravated to mild AR.

In the severe AVP group, for the 2 patients with trivial pre-procedural AR, 1 patient disappeared and 1 patient remained unchanged after procedure. At the end of follow-up, the degree of AR remained unchanged in both patients. In 5 patients with severe AVP without AR before procedure, 1 patient developed mild post-procedural AR. At the end of follow-up, the patient (20%) developed to mild AR and another patient developed trivial AR.

### 3.4. Arrhythmia

As reported in Table 3, only 1 patient (0.61%), with mild AVP, experienced a transient complete atrioventricular block during the procedure, which was eliminated after intravenous injection of atropine and dexamethasone.

Four patients (6.8%) developed post-procedural arrhythmia in mild AVP, including junctional tachycardia in 3 patients and complete right bundle branch block in 1 patient. Eight moderate AVP patients (10%) developed post-procedural arrhythmias, including junctional tachycardia in 2 patients, complete right bundle branch block in 1 patient, incomplete right bundle branch block in 3 patients, and left anterior bundle branch block in 2 patients. There was no post-procedural arrhythmia in patients with severe AVP. All patients with junctional tachycardia recovered after 2–6 days of treatment with methylprednisolone, and without recurrence at follow-up. More than half (57.1%) of the patients with various bundle branch blocks recovered at the 1-month follow-up.

During the follow-up period, 1 patient developed ventricular extrasystole and 8 patients developed bundle branch block, all of which were asymptomatic, and half of which recovered at the end of the follow-up. None of these patients developed complete atrioventricular block or other severe arrhythmias requiring medication, pacemaker implantation, or surgical treatment.

### 3.5. Residual Shunt

In the mild, moderate, and AVP groups, there were 4 (6.8%), 4 (5.0%), and 1 (14.3%) patients with residual shunt, respectively, after the procedure (Table 3). At the end of follow-up, these numbers decreased to 2 (3.6%), 2 (2.8%), and 1 (14.3%) patients with residual shunts in the mild, moderate, and severe AVP groups, respectively. All residual shunts were trivial or mild and did not require transcatheter or surgical treatment.

### 3.6. Follow-Up

The median follow-up time was 70 months (range, 16–91 months). Follow-up data were available for 137 (93.8%) patients, as nine patients (6.2%) were lost to follow-up. Most patients lost to follow-up were due to changes in contact information.

**Figure 3:**
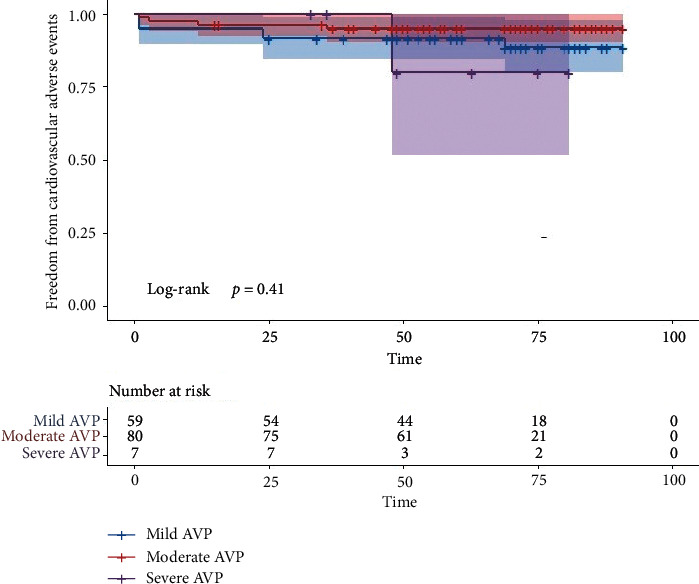
Survival rates without cardiovascular adverse events for 6 years among three degrees of aortic valve prolapse.

No hemolysis, cardiac arrest, shock, more than moderate AR, more than moderate residual shunt, surgical removal of device, implantation of pacemaker due to arrhythmia, or other complications requiring surgical treatment occurred during the follow-up period. Survival rates without cardiovascular adverse events for 6 years among three degrees of AVP are shown in Figure 3.

## 4. Discussion

The optimal time to close VSD with AVP is controversial. Due to the persistent existence of siphon of the VSD shunt, both the incidence of AVP and the possibility of AR increase with age. It is common belief that once AVP appears, VSD should be closed as soon as possible to avoid further aggravation of AVP and the occurrence of AR [7, 13, 14]. In the past, VSD with AVP was mostly treated by surgery. However, with the continuous development of transcatheter technology recently, the device type and material have improved. Various studies have shown that transcatheter occlusion is safe and effective, even in VSD with AVP [7, 8]. Chen and colleagues reported that the success rate of VSD combined with AVP was as high as 96.9% [7]. In this study, the success rates of mild and moderate AVP were 93.7% and 89.9%, respectively; these rates are similar to those seen in this study. Therefore, transcatheter occlusion is feasible for VSD patients with mild and moderate AVP.

AR is one of the most common complications after transcatheter occlusion and may be caused by aortic valve injury resulting from a guidewire or occluder-related injury [15–17]. At the end of follow-up in this study, 2 patients (1.5%) aggravated to moderate AR, and 3 patients (2.2%) had new-onset mild AR. None of the patients had significant effects on ventricular size and cardiac function, and none required therapy. Previous studies reported that AR in VSD with AVP was alleviated or disappeared after transcatheter occlusion [18]. At the end of follow-up in this study, AR disappeared in 17 patients (12.4%) and decreased in 1 patient (0.7%), which was consistent with the literature [18]. The reason may be that the shape of the prolapsed right coronary valve gradually returns to normal [7]. Although Topcuoglu et al. have reported late severe AR and right aortic valve perforation during long-term follow-up, surgical removal of the occluder and repair of VSD are needed [16, 17]. It is worth noting that late AR also occurs during surgical repair of VSD. Aortic valve deformation, AR, and postprocedural residual shunt are risk factors for the occurrence or progression of AR [16]. Therefore, regular examination is necessary to detect AR early after transcatheter occlusion. For patients who have continuous aggravation of AR or valve perforation, it is generally considered that the valve is likely to be affected by the occluder. It is recommended that the occluder be removed by early surgery to avoid difficult removal due to adhesion between the occluder and the surrounding tissue.

Arrhythmia is another common complication, mainly including complete atrioventricular block, bundle branch block, ventricular extrasystole, and junctional tachycardia. The most serious arrhythmia is complete atrioventricular block, with an incidence of 1% to 5% [19–21]. Those who cannot recover after treatment with methylprednisolone or temporary pacemaker need permanent pacemaker treatment [22]. In this study, 1 patient (0.68%) developed complete atrioventricular block immediately after device closure, but recovered after treatment with methylprednisolone, and there was no recurrence during the follow-up. Gianfranco et al. reported the occurrence of late complete atrioventricular block [20, 23]; however, this was not seen in this study. The mechanism of complete atrioventricular block is not clear. It may be related to the stimulation of the conduction bundle by the catheter guidewire, mechanical compression of the occluder, long-term wear of the occluder, tissue edema around the occluder, formation of scar block, or persistent inflammatory reaction [20, 24].

Accurate pre-procedural evaluation of VSD and AVP and reasonable occluder selection are the key to a successful procedure. Pre-procedural transthoracic echocardiography and intra-procedural left ventriculography complement each other, which helps to avoid underestimating the true size of VSD due to partial occlusion of the prolapsed right coronary valve, resulting in inappropriate selection of the occluder. Aortic root angiography and intra-procedural transthoracic echocardiography should be performed before and after occlusion to help clarify AVP and the effect of the occluder on the aortic valve and the residual shunt. The occluders used in this study included symmetrical, eccentric, and ADO-II occluders. According to the experience of our center, when VSD is close to the aortic valve, an ADO-II occluder or eccentric occluder can be used. Symmetrical occluders are suitable for VSD with aneurysms. The softness of the ADO-II occluder can avoid injury to the aortic valve caused by the occluder [15]. The upper edge of the left ventricular disk of the eccentric occluder is 0.5 mm larger than the waist, which can prevent damage to the aortic valve. However, the use of an eccentric occluder has been reduced increasingly in recent years, due to the risk of complete left bundle branch block [25, 26].

### 4.1. Clinical Implications

With the rapid development of transcatheter techniques and instruments, transcatheter occlusion of pVSD was constantly being tried [7, 8], while the efficacy and safety of pVSD with AVP remain controversial. The present study indicates that transcatheter occlusion is a safe and effective treatment for patients with pVSD and AVP.

### 4.2. Limitations

Although transcatheter occlusion of pVSD with mild and moderate AVP is safe and efficient in long-term follow-up, this study was a single-center, retrospective study; these are some limitations of this study. Multicenter, large sample, prospective studies with longer follow-up results are still needed to determine the safety and efficacy of transcatheter occlusion. Furthermore, the selection of occluders in pVSD with AVP should be clarified in the future.

## 5. Conclusion

pVSD with mild and moderate AVP has a high success rate and few complications; therefore, it is safe and efficient for transcatheter occlusion. However, pVSD with severe AVP has a low success rate and often leads to AR; transcatheter occlusion is not recommended.

## Figures and Tables

**Table 1:** Patient characteristics.

Variables	
Gender, *n* (%)	
Male	97 (59.1%)
Female	67 (40.9%)
Age (mos.), median [IQR]	40.00 (30.00, 62.75)
Weight (kg), mean ± SD	16.94 ± 9.02
Height (cm), mean ± SD	102.84 ± 19.75
Size of VSD by TTE (mm), mean ± SD	3.75 ± 1.35
TPG (mmHg), mean ± SD	71.96 ± 13.78
Degree of TR, *n* (%)	
Trivial	40 (24.4%)
Mild	55 (33.5%)
Moderate	5 (3.0%)

VSD, ventricular septal defect; TTE, transthoracic echocardiography; TPG, transseptal pressure gradient; TR, tricuspid regurgitation.

**Table 2:** Radiographic data for different degrees of aortic valve prolapse.

Variables	Mild AVP	Moderate AVP	Severe AVP
Number	63	89	12
Size of VSD, mean ± SD	3.85 ± 1.49	3.65 ± 1.29	3.92 ± 1.13
QP/QS	1.85 ± 0.54	1.79 ± 0.60	1.73 ± 0.61
MPAP (mmHg), mean ± SD	18.94 ± 5.78	17.00 ± 4.56	14.83 ± 4.20
Aneurysm, *n* (%)	26 (41.3)	45 (50.6)	5 (41.7)
Noncoronary valve prolapse, *n* (%)	24 (38.1)	41 (46.1)	6 (50.0)
Degree of AR, *n* (%)			
Trivial	4(6.3)	7 (7.9)	4 (33.3)
Mild	4 (6.3)	21 (23.6)	2 (16.7)
Moderate	0 (0)	1 (1.1)	0 (0)

AVP, aortic valve prolapse; VSD, ventricular septal defect; Qp/Qs, ratio of pulmonary and systemic blood flow; MPAP, mean pulmonary artery pressure; AR, aortic regurgitation.

**Table 3:** Procedural results of different degrees of aortic valve prolapse.

Variables	Mild AVP	Moderate AVP	Severe AVP
Success rate, *n* (%)	59/63 (93.7)	80/89 (89.9)	7/12 (58.3)
Size of the occluder (mm), mean ± SD	6.41 ± 1.40	6.52 ± 1.26	6.00 ± 1.29
Type of the occluder, *n* (%)			
Symmetrical VSD occluder	44 (74.6)	50 (62.5)	1 (14.3)
Eccentric VSD occluder	13 (22.0)	28 (35)	4 (57.1)
Amplatzer Duct Occluder II	2 (3.4)	2 (2.5)	2 (28.6)
Degree of AR, *n* (%)			
Trivial	14 (23.7)	21 (26.3)	1 (14.3)
Mild	1 (1.7)	2 (2.5)	1 (14.3)
Moderate	0 (0)	0 (0)	0 (0)
Residual shunt, *n* (%)	4 (6.8%)	4 (5.0%)	1 (14.3%)
Arrhythmia, *n* (%)			
CAVB	1 (1.7)	0 (0)	0 (0)
LABBB	0 (0)	2 (2.5)	0 (0)
CRBBB	1 (1.7)	1 (1.3)	0 (0)
IRBBB	0 (0)	3 (3.8)	0 (0)
Junctional tachycardia	3 (5.1)	2 (2.5)	0 (0)

VSD, ventricular septal defect; AR, aortic regurgitation; CAVB, complete atrioventricular block; LABBB, left anterior bundle branch block; CRBBB, complete right bundle branch block; IRBBB, incomplete right bundle branch block.

## Data Availability

The data used to support the findings of this study are available from the corresponding author upon request.
